# Supplementary material for: Nord Stream methane leaks spread across 14% of Baltic waters
Source: Nat Commun. 2025 Jan 15;16:281. doi: 10.1038/s41467-024-53779-0 (PMC11735640; doi:10.1038/s41467-024-53779-0)
Supplement: Supplementary file 1 — Supplementary Information [file 41467_2024_53779_MOESM1_ESM.pdf]

Supplementary information to: Nord Stream methane leaks spread across 14% of Baltic waters

Martin Mohrmann[1,2*], Louise C. Biddle[1,2], Gregor Rehder[3], Henry C. Bittig[3], Bastien Y. Queste[2]

[1*]Voice of the Ocean Foundation, Skeppet Ärans Väg 19, Västra Frölunda, 426 71, Sweden.
[2]Department of Marine Science, University of Gothenburg, Box 463, Göteborg, 405 30, Sweden.
[3]Leibniz-Institute for Baltic Sea Research, Seestrasse 15, Rostock, D-18119, Germany.

*Corresponding author(s). E-mail(s): martin.mohrmann@voiceoftheocean.org;
Contributing authors: louise.biddle@voiceoftheocean.org; gregor.rehder@io-warnemuende.de; henry.bittig@io-warnemuende.de; bastien.queste@marine.gu.se;

S1 Bornholm Basin Oceanographic conditions

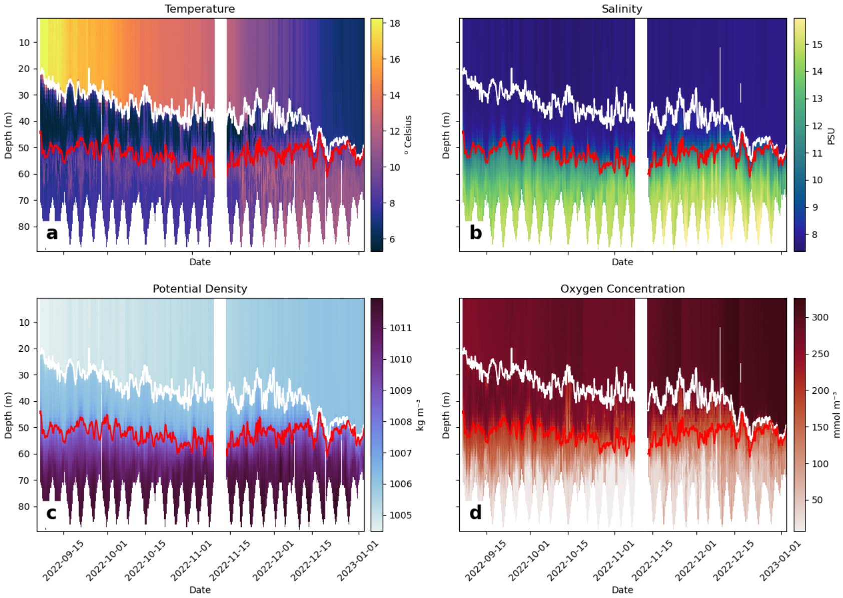

Fig. S1 SAMBA Bornholm Glider observatory. a temperature, **b** salinity, **c** density **d** oxygen concentration. The MLD (potential density threshold method 0.03 g l^{-1}) and halocline (maximum dS/dp gradient) are marked in white and red lines respectively.

S2 Initial Conditions and Model tuning

The first two weeks of simulation after the Nord Stream incidents were run iteratively with different initial amounts of methane, until a good correlation between the initially observed methane concentration and the modeled methane concentration was reached (Figure S2). The gradients agree relatively well for the first week of observations (Figure **??**a), establishing trust in our model results. For the uncertainty estimation of our results, we used the python statsmodels package to calculate a 2 standard deviations range of the slope of an ordinary least squares fit (Figure S2b). To check the robustness of the result, we created similar linear regressions with (1) the surface data of the SOOP system and (2) the whole length of the Glider dataset. These linear regressions resulted in different slopes of 0.80 and 0.94 respectively so we added these deviations (apparent underestimation of the modeled dissolved methane concentrations of up to 20%) to our uncertainty range. Moreover, the model was driven by two different engineering estimates of methane escaping the pipelines (PBREAK

and CATHARE), which were tested in separate model runs and resulted in a difference of dissolved methane of about 6% (not shown). Finally, each of the engineering models had their own uncertainty boundaries. We add all the above mentioned components of the uncertainty analysis to derive uppler- and lower boundary estimations of the total dissolved methane.

To keep the model simple, we assumed an initially homogeneous depth distribution, in the horizontal dimension the methane is initialized following a normal distribution with radius of 5 km around the leak sites. We choose this relatively wide initial distribution (in comparison to Dissayanake et al., 2023) to account for eventual turbulent mixing, water recirculation and uncertainties in the model currents and initial conditions. Bubble plume dynamics at sites of strong methane release are difficult to predict [1]. However, the deep thermocline and the trough-like structure of the Bornholm basin reduces the effect of the uncertainty of dissolution rates as function of depth.

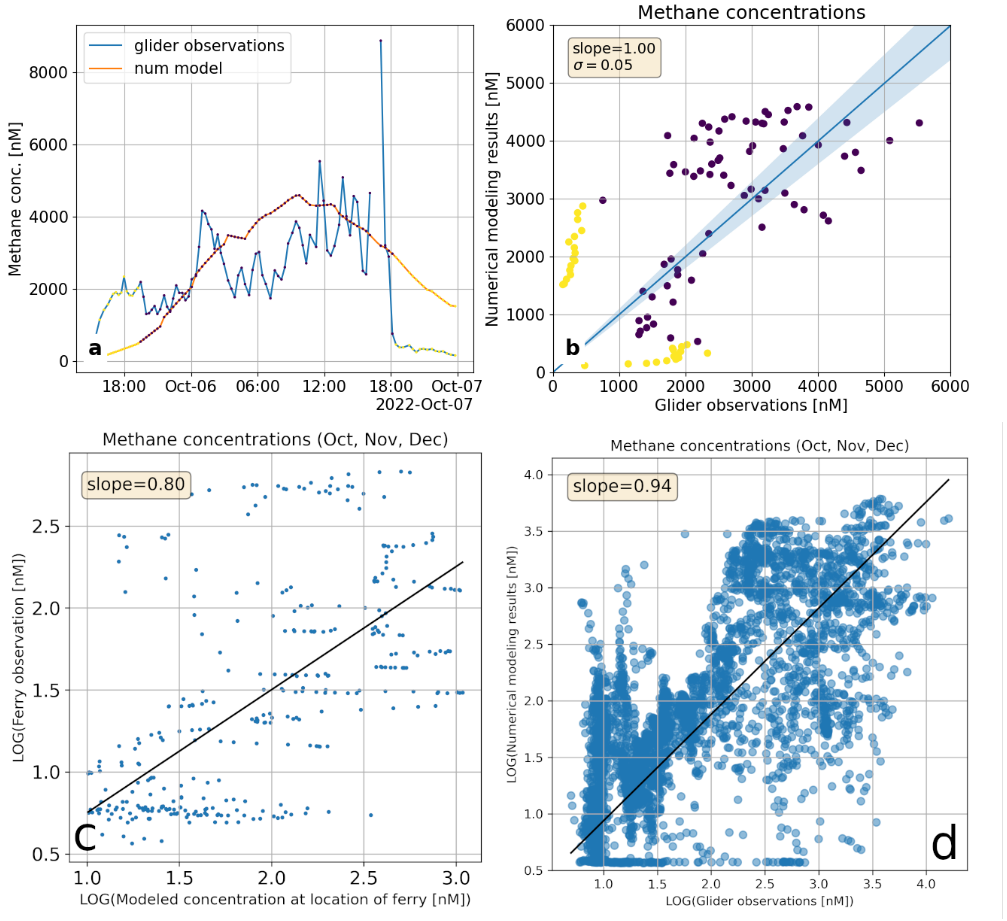

Fig. S2 **a** Glider observations and model output for the first 28 h after the glider deployment. **b** Linear regression of glider observations with numerical model, after tuning the initial release of methane. Yellow data points were filtered out before linear regression due to spatio-temporal miss-match between model and observations. **c** Linear regression of SOOP surface observations with model results. **d** Linear regression of full time series of Glider data with model results. **c** and **d** were used for uncertainty estimation only. They were log-scaled to reduce heteroscedasticity, a statistical issue when comparing data across multiple orders of magnitude.

S3 Oxidation rates

Dissolved methane concentrations can decrease by advection/diffusion, outgassing or oxidization. While the former processes are well constraint by physical equations, the oxidization rate has a large uncertainty. Maximum oxidation rates in the Baltic Sea under natural conditions are 0.0079 and 0.0022 d^{-1} for the Landsort Deep and Gotland Deep in the presence of 200 and 799 nM CH4 respectively [2]. These concentrations are the same order of magnitude that we measure throughout the water column after

the Nord Stream leaks. However, the continued presence of natural methane maxima in the deepest parts of the Baltic Sea allowed for well established populations of methanotrophs, which might not be the case for the Nord Stream leak sites. Moreover, the above mentioned rate constants are maxima, thus we assume similar or lower rate constants on average. [3] use a rate constant of 0.035 d^{-1} in their Nord Stream study. This rate is the maximum rate based on a site in the North Sea where high methane concentrations were present for nearly 30 years without interruption and hence, can be seen as an upper estimate.

Here we conduct a sensitivity study with constant rate constants of 0.035 d^{-1}, 0.0175 d^{-1} and 0.0035 d^{-1}. This translates to turnover times of 29, 127 and 455 days respectively. Compared to the average outgassing rate of 0.03 d^{-1}, oxidation is likely less effective in removing methane from the water during the weeks following the leaks. Using the highest oxidation rate of 0.035 d^{-1}, our model looses the observed spikes of high methane concentrations (>100 nM) after mid-November, thus we consider this oxidisation rate as unlikely. A rate of 0.0175 d^{-1} permits for some patches of increased methane concentrations, though less than observed while a rate of 0.0035 d^{-1} changes our original model output relatively little, because the oxidation rate is small compared to outgassing and advection/dilution processes.

Only the lowest oxidation rate 0.0035 d^{-1} has some of these spikes left, and can therefore be considered as most realistic.

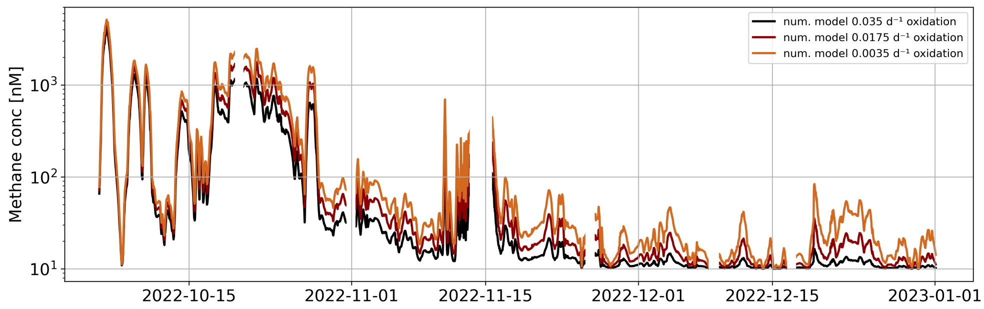

Fig. S3 Sensitivity test for oxidation rates.

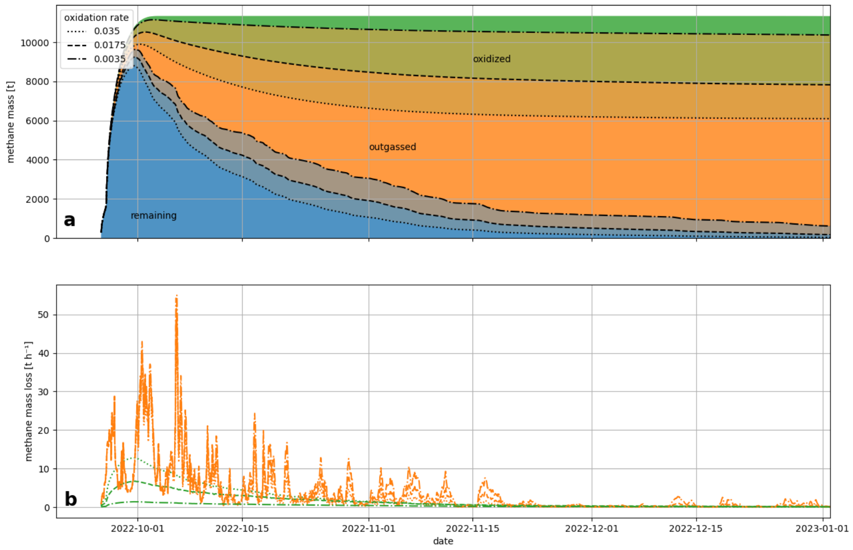

Fig. S4 Mass balance for different oxidation rates. a Depending on the oxidation rate, different mass fractions of dissolved methane remain in the water, outgass or oxidize. **b** Rate of methane loss caused by outgassing (orange) and oxidation (green) for different oxidation rates (see linestyle legend in a)

S4 Methane exposure of Marine Protected Areas

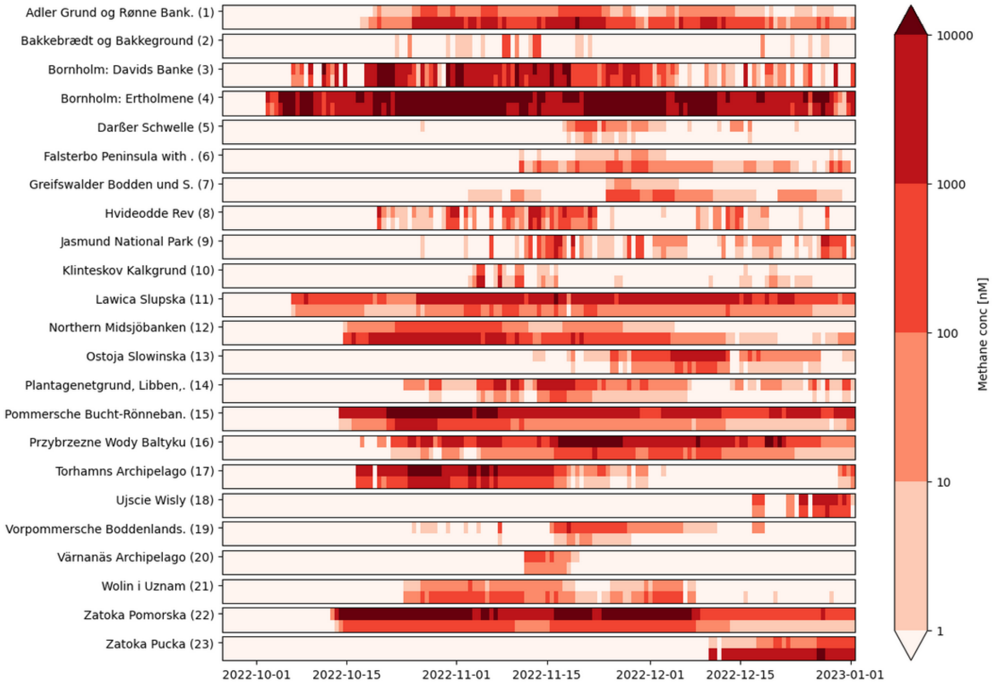

Fig. S5 Marine protected area methane concentration exposures. MPA specific maximum (upper part of the bar) and volume-mean (lower part of the bar) methane concentrations.

References

[1] Leifer, I., Solomon, E., Schneider von Deimling, J., Rehder, G., Coffin, R., Linke, P.: The fate of bubbles in a large, intense bubble megaplume for stratified and unstratified water: Numerical simulations of 22/4b expedition field data. Marine and Petroleum Geology **68**, 806–823 (2015) https://doi.org/10.1016/j.marpetgeo.2015.07.025 . The 224b Study Investigations of the Geologic, Water-Column, and Atmospheric Phenomena of a Persistent North Sea Gas Blowout

[2] Jakobs, G., Rehder, G., Jost, G., Kießlich, K., Labrenz, M., Schmale, O.: Comparative studies of pelagic microbial methane oxidation within the redox zones of the Gotland Deep and Landsort Deep (central Baltic Sea). Biogeosciences **10**(12), 7863–7875 (2013) https://doi.org/10.5194/bg-10-7863-2013

[3] Dissanayake, A.L., Gros, J., Drews, H.J., Nielsen, J.W., Drews, A.: Fate of Methane from the Nord Stream Pipeline Leaks. Environmental Science & Technology Letters **10**(10), 903–908 (2023) https://doi.org/10.1021/acs.estlett.3c00493
